# Where Are the Newly Diagnosed HIV Positives in Kenya? Time to Consider Geo-Spatially Guided Targeting at a Finer Scale to Reach the “First 90”

**DOI:** 10.3389/fpubh.2021.503555

**Published:** 2021-04-23

**Authors:** Anthony Waruru, Joyce Wamicwe, Jonathan Mwangi, Thomas N. O. Achia, Emily Zielinski-Gutierrez, Lucy Ng'ang'a, Fredrick Miruka, Peter Yegon, Davies Kimanga, James L. Tobias, Peter W. Young, Kevin M. De Cock, Thorkild Tylleskär

**Affiliations:** ^1^Centre for International Health, University of Bergen, Bergen, Norway; ^2^Division of Global HIV and TB, U.S Centers for Disease Control and Prevention, Nairobi, Kenya; ^3^National AIDS and Sexually Transmitted Infections (STI) Control Program, Ministry of Health, Nairobi, Kenya; ^4^Health Population and Nutrition, United States Agency for International Development, Nairobi, Kenya; ^5^Division of Global HIV and TB, U.S Centers for Disease Control and Prevention, Atlanta, GA, United States

**Keywords:** UNAIDS 90-90-90 Fast-Track targets, HIV testing, spatial auto-correlation, hotspots, country operational plans, Kenya

## Abstract

**Background:** The UNAIDS 90-90-90 Fast-Track targets provide a framework for assessing coverage of HIV testing services (HTS) and awareness of HIV status – the “first 90.” In Kenya, the bulk of HIV testing targets are aligned to the five highest HIV-burden counties. However, we do not know if most of the new HIV diagnoses are in these five highest-burden counties or elsewhere.

**Methods:** We analyzed facility-level HTS data in Kenya from 1 October 2015 to 30 September 2016 to assess the spatial distribution of newly diagnosed HIV-positives. We used the Moran's Index (Moran's I) to assess global and local spatial auto-correlation of newly diagnosed HIV-positive tests and Kulldorff spatial scan statistics to detect hotspots of newly diagnosed HIV-positive tests. For aggregated data, we used Kruskal-Wallis equality-of-populations non-parametric rank test to compare absolute numbers across classes.

**Results:** Out of 4,021 HTS sites, 3,969 (98.7%) had geocodes available. Most facilities (3,034, 76.4%), were not spatially autocorrelated for the number of newly diagnosed HIV-positives. For the rest, clustering occurred as follows; 438 (11.0%) were HH, 66 (1.7%) HL, 275 (6.9%) LH, and 156 (3.9%) LL. Of the HH sites, 301 (68.7%) were in high HIV-burden counties. Over half of 123 clusters with a significantly high number of newly diagnosed HIV-infected persons, 73(59.3%) were not in the five highest HIV-burden counties. Clusters with a high number of newly diagnosed persons had twice the number of positives per 1,000,000 tests than clusters with lower numbers (29,856 vs. 14,172).

**Conclusions:** Although high HIV-burden counties contain clusters of sites with a high number of newly diagnosed HIV-infected persons, we detected many such clusters in low-burden counties as well. To expand HTS where most needed and reach the “first 90” targets, geospatial analyses and mapping make it easier to identify and describe localized epidemic patterns in a spatially dispersed epidemic like Kenya's, and consequently, reorient and prioritize HTS strategies.

## Introduction

During 2016, it was estimated that between 1.6 million and 2.1 million people were tested positive for HIV and learned their status worldwide (1). Human immunodeficiency virus (HIV) testing and knowledge of status are the first steps in the UNAIDS 90-90-90 targets-based HIV epidemic control strategy. The so-called “Fast Track” targets aim for 90% of persons living with HIV (PLHIV) knowing their HIV status, 90% of people with diagnosed HIV infection receiving antiretroviral therapy (ART), and 90% of those on ART virally suppressed by 2020 (2). These targets were revised in 2017 to 95-95-95 for the same care and treatment indicators to be attained by 2030 (3). This translates to a viral load suppression rate of 73 or 85% of all PLHIV (for the 90-90-90 and 95-95-95 “Fast Track” targets, respectively). Measuring the Fast Track targets provides a framework for assessing coverage of HIV testing services (HTS), linkage to care for those diagnosed with HIV, and viral load suppression for those on ART. Achieving the “first 90” subsequently affects the treatment numbers and has become a basis in determining subsequent targets along the treatment cascade.

The focus on HIV programming to the “right places” and reprogramming of HIV diagnoses, care, and treatment activities to the highest-burden areas has been the cornerstone of targeting and resource allocation in recent years (4). It is expected that with epidemic control, there will be fewer than 500,000 annual newly HIV-infected persons globally (3). Hence, it will be increasingly hard to find new HIV diagnoses without properly planned location-based HTS strategies. To achieve the “first 90,” it is necessary to identify the best HTS strategies that help identify most HIV-infected persons at the lowest cost, including event and location-based testing (3). However, in a generalized epidemic, identifying the newly diagnosed HIV-infected persons and putting them on treatment are increasingly difficult without efficient and context-appropriate strategies.

Kenya has a generalized HIV epidemic with a prevalence among 15–49-year-olds estimated to be 5.9% in 2015 (5) and 4.9% in 2018 (6). In 2017, Kenya was ranked 3rd among eastern and southern African countries in the estimated number of new HIV infections among adults aged 15 years and older (7). Geospatial variation of HIV prevalence is wide-ranging from 21.0% in Siaya county to the lowest 0.4% in Wajir County and an approximated 52,000 new infections across all ages (6). Accordingly, the focus of HTS targeting to reach more newly infected persons is in the five highest HIV-burden counties: Nairobi, Homabay, Kisumu, Siaya, and Migori, collectively accounting for over 40% of the estimated PLHIV in Kenya. However, it may not be true that most of the newly diagnosed HIV-infected persons are in the highest-burden counties since geographic disparities may exist within larger health planning geographic units. Additionally, there is the possibility of existing pockets of hyper-epidemics in low burden counties and regions.

Previous analysis of population-based household survey data in Kenya has shown that clusters of high rates of HIV infections may exist even in low burden counties (8). Examining the HIV burden without regard to arbitrary physical boundaries that may confine populations by ethnicity, culture, human settlements, and natural resources provides an opportunity for equitable resource allocation. Such analyses help us to unmask local patterns that go beyond the traditional data aggregation methods. They would help focus and prioritize HTS strategies to reach the “first 90” and monitoring the HIV epidemic (9). Our analyses' objective was to provide a spatial presentation of newly diagnosed HIV-infected persons in Kenya and assess whether the spatial patterns match HTS resource planning. We illustrate how such analyses can be used to identify spatial clusters with many newly diagnosed HIV-infected persons to provide a finer-scale geographical context for HTS programming.

## Materials and Methods

### Setting and HTS Planning Context

**Figure 1:**
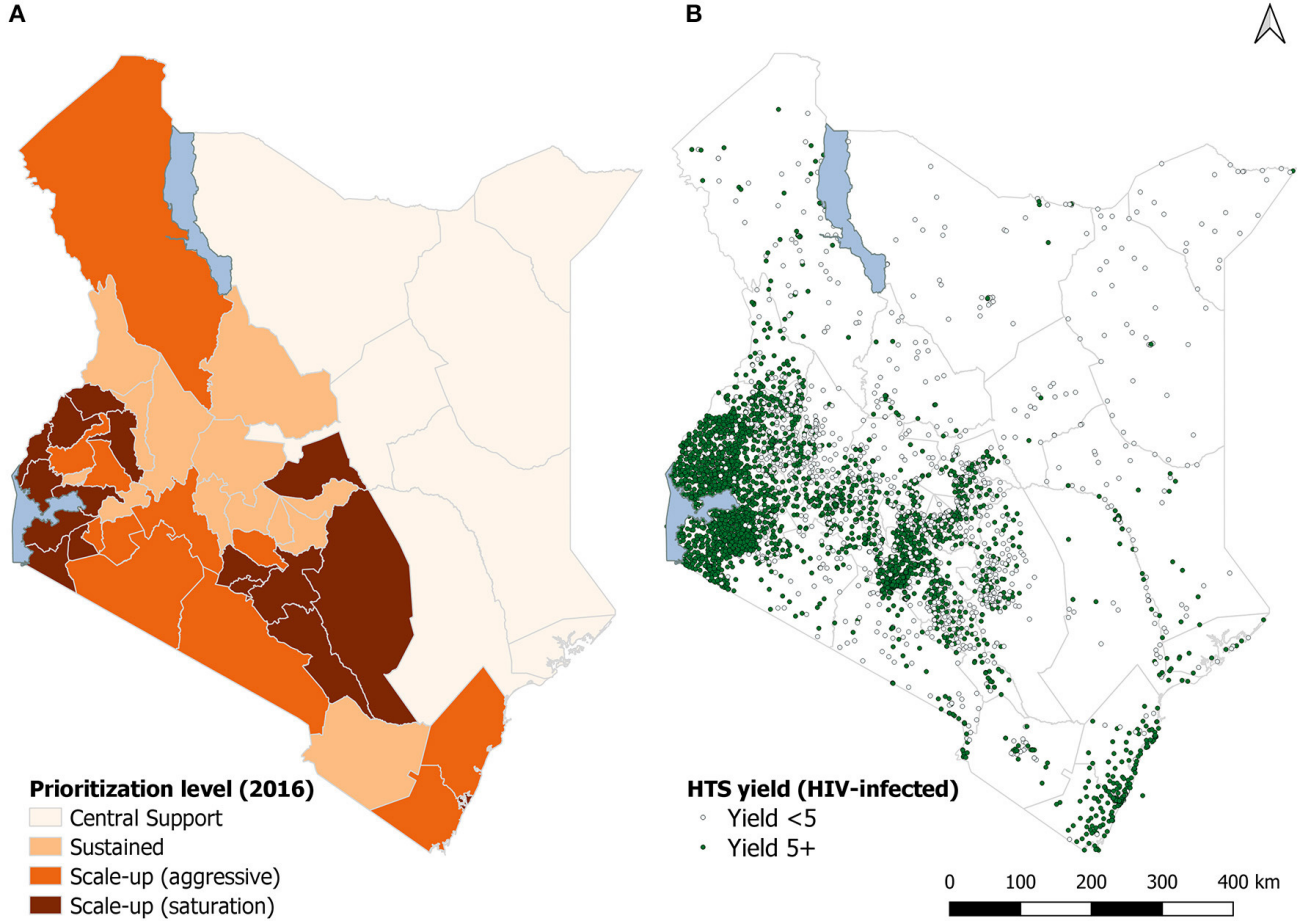
County classification in the country operational plan **(A)** and reporting sites **(B)**, Kenya 2016. **(A)** of the figure shows the country operational plan county-classification. **(B)** shows the sites reporting HIV testing by the yield of HIV-infected persons.

Kenya is a geographically diverse country in East Africa with a population density of 85.1 people per sq. km in 2016 (10). It has 47 counties that form the structure for a decentralized system of government. In 2016, the five highest-HIV burden counties were: Nairobi, Homabay, Kisumu, Siaya, and Migori (11). In the same year, over 3,900 facilities provided HTS by preventing mother to child transmission (PMTCT), TB clinics, comprehensive care centers (CCCs) for HIV care, and outpatient and inpatient departments. Implementation of HIV program supported through the US President's Emergency Plan for AIDS Relief (PEPFAR) is guided by the country operational plan (COP). This is a planning process involving multiple partners in HIV programming and funding. In the Kenya planning cycle of 2016, counties were classified into four categories: scale-up to saturation; scale-up aggressive; sustained; and sustained centrally supported for commodities only. Thus, PEPFAR efforts focused on the five highest-burden counties and intensified support in 11 additional counties. The aim was to reach saturation (at least 80% of PLHIV diagnosed and knowing their HIV status) in 16 counties by the end of the fiscal year 2017. For the other 11 high burden counties, COP 2016 proposed to continue aggressive scale-up. Within the remaining 20 counties, 13 were classified as sustained facility-level support and seven for centrally-supported commodities (12) (Figure 1).

### HTS Data Source and Aggregation

All HTS facilities report the number tested and the number newly diagnosed with HIV to the Ministry of Health through the District Health Information System version 2 (DHIS2) platform <https://hiskenya.org/>. Data were aggregated at the facility-level and counties for analyses. Our hypothesis was that HIV-infected persons were distributed randomly within geolocations. In addition to the COP classification, we created county categories by standardizing the burden of HIV as the percentile distribution of the number of PLHIV per 100,000 population within a county.

### Geo-Data

Shape polygons were sourced from various public repositories: projections from <http://www.epsg.org/> and other GIS data from <http://192.156.137.110/gis/search.asp>, <https://www.wri.org/resources/data-sets/kenya-gis-data> – all these data are freely available and their access and use are covered under the creative commons license CC BY 4.0. For all the projections, we used the world geodetic system (WGS) 1984 coordinate reference system. For point-pattern data, we obtained the master facility list that contains latitude and longitude coordinates of all public health facilities in the country from the Kenya Ministry of Health as a comma separated values (CSV) file format and imported this into the GIS software as a delimited text layer for analysis and mapping.

### Measures and Summary Analyses

Our primary outcome of interest was the count of new HIV diagnoses from 1 October 2015 to 30 September 2016 by each health facility and further aggregated at the county level. These data are routinely collected and submitted centrally as part of HIV program monitoring in Kenya. HIV infections were diagnosed using the national HIV testing algorithm (13). The denominator was the number tested in each facility for the same reporting period. To compare the number of newly diagnosed HIV-infected persons across county categories as defined in the Kenya 2016 COP and using the HIV burden classified using percentiles, we used Kruskal-Wallis equality-of-populations non-parametric rank test.

### Geo-Statistical Methods

In the first approach, we used local Moran's I in ArcGIS™ version 10.4 to analyze hotspots of the number of newly diagnosed HIV-infected persons at site level hence classifying sites as having no-clustering (random distribution) or autocorrelated neighbors using the four categories: HH, HL, LH, and LL. In these categories, hotspots are represented by an H, while an L denotes low spots. In this analysis, we used all the geocoded facilities (*n* = 3,933). In the second approach, we used Kulldorff spatial-scan Poisson model implemented in the software SATScan™ ver. 9.6 to detect whether counts of newly diagnosed HIV-infected persons were randomly distributed over space or clustered in the detectable pattern(s). We considered the number tested for HIV as the population denominator in all the geocoded facilities. We set a circular scan window of 50 kilometers radius scan window that looped over geocoded locations collectively containing HIV-infected persons out of the tested population, assuming the population's proportion at risk to be the default ≤ 50%. We restricted cluster detection to those with at least 10 infected persons and high-rate clusters as those with a relative risk ≥ 1.0. We reported significant Standard Monte Carlo *p*-values for all identified clusters after looping through a maximum of 999 iterations. A cluster was considered statistically significant when its log-likelihood ratio was greater than the critical value. Finally, we compared the hotspots spatial scan output map to local Moran's I autocorrelation maps. For all analyses, statistical significance was considered at *p*-values < 0.05.

## Results

### Do Locations for New HIV Diagnoses Match HIV Burden?

**Figure 2:**
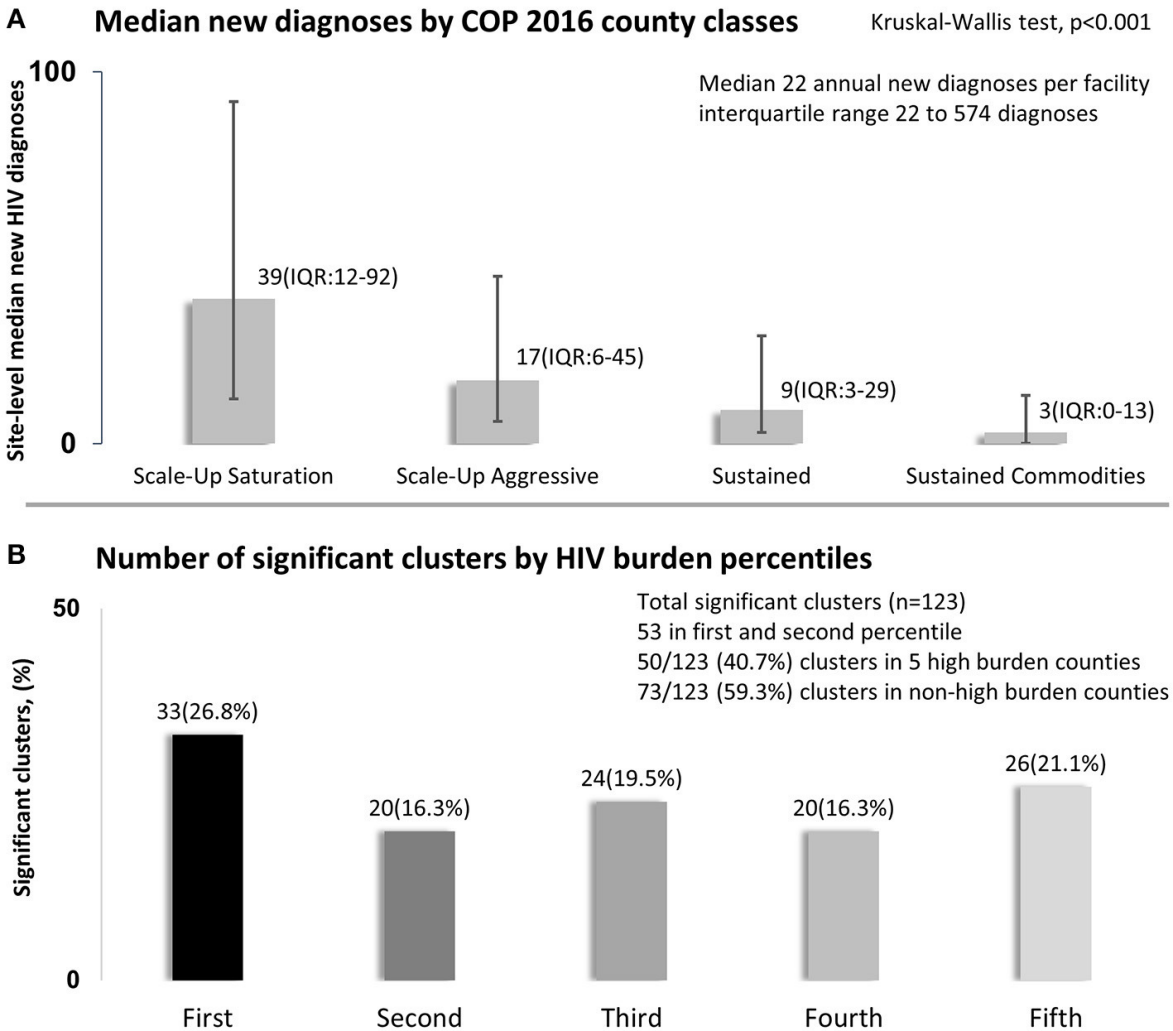
New HIV diagnoses in relation to country operational plan **(A)** and by HIV burden percentiles **(B)**, Kenya 2016. **(A)** of the figure shows median number of annual site-level new HIV diagnoses per county classification. **(B)** shows percent distribution significant 123 clusters by county and HIV burden classes standardized per 100,000 population and classified using percentiles.

Median HIV case identification per facility was 22 new diagnoses in a year [interquartile range (IQR) 22–574]. The number of newly diagnosed HIV-infected persons varied by county classification in Kenya's country operational plan (Figure 2A). Facilities in counties where services were planned for scale-up to saturation had the highest median number of newly diagnosed HIV-infected persons [39 new HIV diagnoses per facility (IQR 12–92)]. However, facilities with the lowest number of newly diagnosed HIV-infected persons were in centrally supported counties, *p* < 0.001. We identified 166 clusters of varying sizes from all geocoded facilities that had high counts of HIV-infected persons. Out of 166 clusters identified, 123 (74%) clusters had a significantly high number of newly diagnosed HIV-infected persons. Less than half of those, 53/123 (43.1%) were in the first and second percentile of HIV burden in Kenya (Figure 2B). Similarly, less than half 50/123 (40.7%) clusters were in five high burden counties, and most, 73/123 (59.3%), were not in the five highest HIV burden counties.

### Site-Level Spatial Auto-Correlation

**Figure 3:**
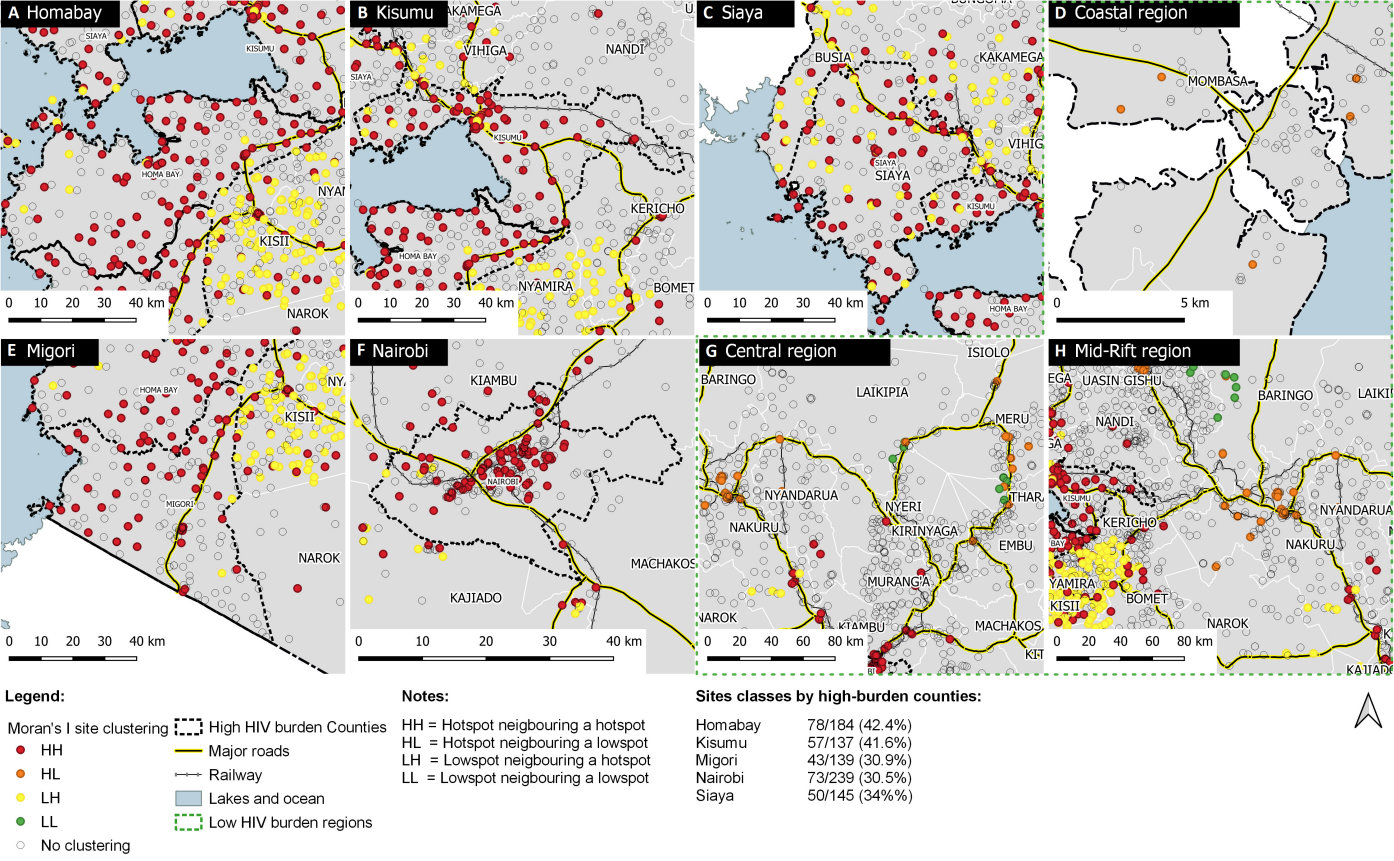
Spatial clustering of newly diagnosed HIV-infected persons in five high HIV-burden counties and low-burden region, Kenya 2016. **(A–C,E,F)** show sites within the five high HIV burden counties according to Moran's I local auto-correlation clustering classes. **(D,G,H)** provide a contrast of sites distributed according to clustering but for low HIV burden regions spanning across multiple counties. Hotspots are represented by (H) and low spots by an (L) neighboring each other in these combinations HH, HL, LH, and LL.

Of 3,968 sites, 3,933 (99.1%) had geocodes available. Using facility-level data, global Moran's I was 0.023, while the expected index was −0.00025, Z-score 33.9, and *p* < 0.001. Most facilities showed no clustering (3,034, 76.4%); others were grouped as follows: HH (438, 11.0%), HL (66, 1.7%), LH (275, 6.9%), and LL (156, 3.9%). Of the HH sites, 301 (68.7%) were in high HIV-burden counties distributed within each: Homabay with 78/184 (42.4%), Kisumu 57/137 (41.6%), Siaya 50/145 (34.5%), Migori 43/139 (30.9%), and Nairobi 73/239 (30.5%). HH facilities in high burden counties were near water bodies (Homabay, Kisumu, Siaya, and Migori) or within the urban core of a large city (Nairobi), and low HIV-burden areas were near major roads (Figure 3).

In Figures 3A–C,E,F, we have presented site-level clustering for newly diagnosed HIV-infected persons for the five high HIV burden counties. In Figures 3D,G,H, we have contrasted the same analyses for low HIV burden regions. Scales for these map collages are different and present high clustering within a short geographic scale and comparatively high clustering for low HIV-burden areas within a broader scale. In absolute terms, the observed number of HIV-infected persons within significant clusters was 192,608 out of an expected 122,489, representing 57.2% more HIV-infected persons within these clusters.

### Comparison of Spatial-Scan and Auto-Correlations

**Figure 4:**
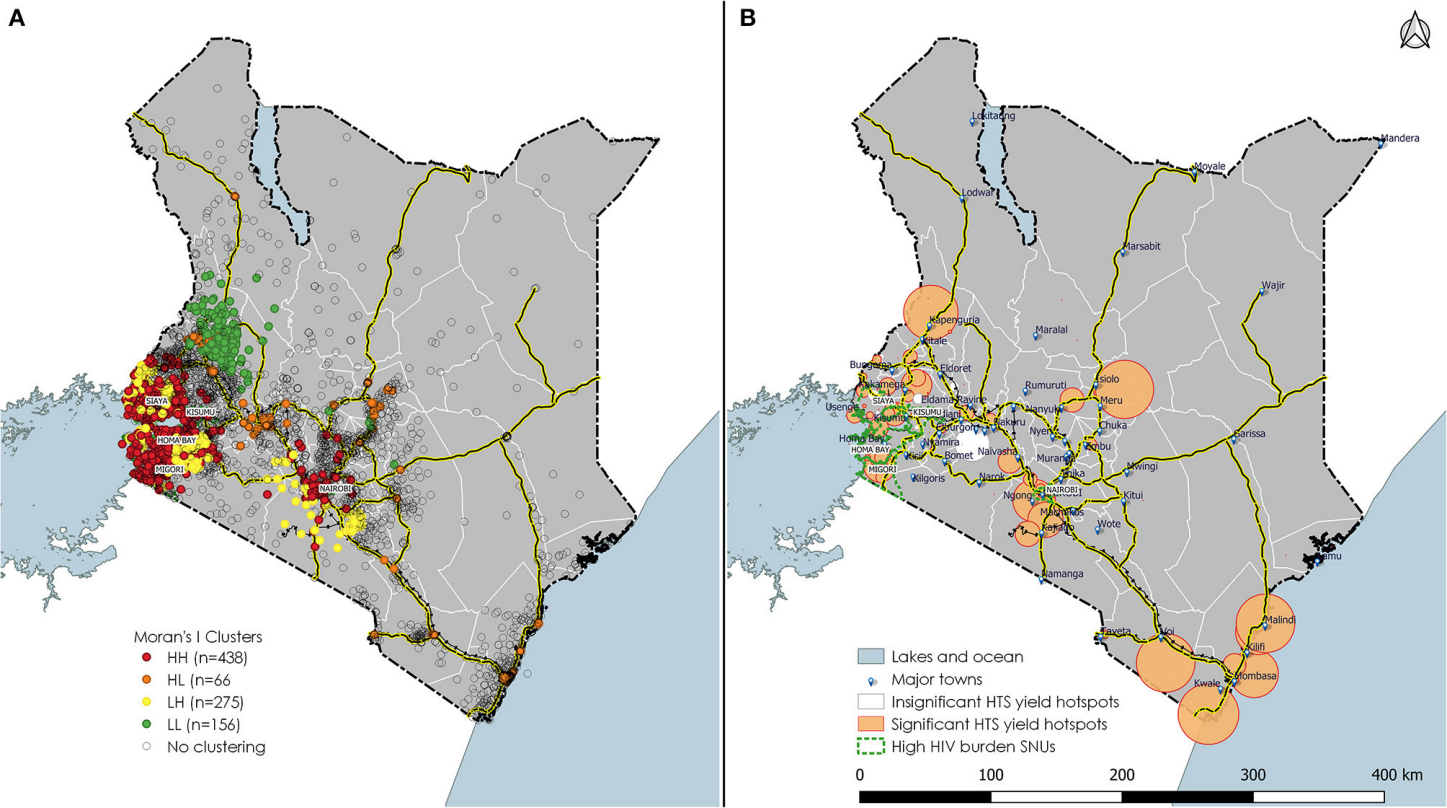
Local Moran's I clustering analyses and Kulldorff spatial-scan analyses of HTS yield taking into account the number tested, Kenya 2016. **(A)** of the figure shows site level auto-correlation and **(B)** shows significant and non-significant clusters identified. Hotspots are represented by (H) and low spots by an (L) neighboring each other in these combinations HH, HL, LH, and LL.

A comparison of facility-level spatial autocorrelation using Moran's I and Kulldorff spatial scan statistics shows similar patterns in western Kenya, along the major Mombasa-Nairobi-Nakuru transport corridor and also around Mt. Kenya, specifically within Meru county, which is a highly productive agricultural area and also known for production of *Catha edulis* commonly referred to as “*Khat*.” Khat is a plant whose leaves are chewed by people for its stimulant action and is a highly-valued export crop. Other clusters were near or within Isiolo town in eastern Kenya and around Nanyuki town in Laikipia county – both towns situated along a major transport corridor to northern Kenya (Figure 4). Bigger clusters were identified at the coastal region in Mombasa, Malindi, Kwale, and Voi towns. We also identified clusters in Kajiado County along the Nairobi-Namanga major highway and toward the Kenya-Tanzania border.

### Performance Against Contextual Resource Allocation

In Table 1, we present sites performance against HTS targets. Overall yield was 2.0% (239,407/ 12,172,947) in 3968 sites. Testing achievement was surpassed by 68.4% (12,172,947/7,229,811).

**Table 1:** HIV testing services performance vs. contextual planning, Kenya 2016.

**County class^**†**^**	**HTS target**	**Offered HTS**	**HIV diagnosed**	**Sites**	**Yield^**‡**^**
Scale-up saturation	4,426,254	7,676,280	165,097	1,973	2.2%
Scale-up aggressive	1,903,610	2,975,476	50,671	1,095	1.7%
Sustained	742,937	1,352,538	21,676	775	1.6%
Sustained	157,010	168,653	1,963	125	1.2%
commodities					
Total	**7,229,811**	**12,172,947**	**239,407**	**3,968**	**2.0%**

† *Classes defined according to COP 2016*.

‡ *Calculated as HIV diagnosed/the number offered HTS*.

A third of the sites (1,350) contributed to new diagnoses in clusters with many new HIV diagnoses (Table 2). Clusters with a high number of newly diagnosed persons had twice the number of positives per 1,000,000 tests than clusters with lower numbers (29,856 vs. 14,172).

**Table 2:** Performance of HIV testing services in higher compared to lower yielding areas, Kenya 2016.

**County class^**†**^**	**HIV diagnosed**	**Offered HTS**	**Sites**	**Yield^**‡**^**
**Higher-yielding areas**				
Scale-up saturation	95,791	3,151,048	789	3.0%
Scale-up aggressive	23,642	819,955	376	2.9%
Sustained	7,323	282,508	178	2.6%
Sustained commodities	577	11,388	7	5.1%
*Sub-total*	*127,333*	*4,264,899*	*1,350*	*3.0%*
**Lower-yielding areas**				
Scale-up saturation	69,306	4,525,232	1,184	1.5%
Scale-up aggressive	27,029	2,155,521	719	1.3%
Sustained	14,353	1,070,030	597	1.3%
Sustained commodities	1,386	157,265	118	0.9%
*Sub-total*	*112,074*	*7,908,048*	*2,618*	*1.4%*
Total	**239,407**	**12,172,947**	**3,968**	**2.0%**

† *Classes defined according to country operational plan 2016*.

‡ *Calculated as HIV diagnosed/the number offered HTS*. *New diagnoses per 1,000,000 tests = 127,333/4,264,899 × 1,000,000 in sites within higher yielding areas = 29,856*. *New diagnoses per 1,000,000 tests = 112,074/7,908,048 × 1,000,000 in sites within lower yielding areas = 14,172*. *The values in italics are sub-totals*.

## Discussion

Where are the newly diagnosed HIV positives in Kenya? About 3 out of 5 of clusters with a high number of newly diagnosed HIV-infected persons were not in the five highest HIV burden counties of Kenya, with a substantial proportion of sites contributing to an increased number of newly diagnosed HIV-infected persons in low HIV burden counties. We found sites with many newly diagnosed HIV-infected persons found in low-burden counties typically situated near transport corridors. Major transport corridors play a role in HIV transmission to relatively rural areas has been described by others (14). Additionally, such sites contributing to clusters with high numbers of HIV-infected persons are often situated near major economic activities such as extensive agricultural activities, fishing, and informal settlements (8), thereby demonstrating the geographical diversity and rurality of HIV.

We interpret our findings in two ways: firstly, we answer the question of whether higher numbers of newly diagnosed HIV-infected persons are found in the highest-burden counties, and secondly, we qualitatively examine the role of transport infrastructure in the geographic distributions of the newly diagnosed.

Does the number of newly diagnosed HIV-infected persons follow the HIV burden? The common belief in HIV programming is that many newly diagnosed HIV-infected persons would be within high HIV-burden counties instead of low-burden counties. Our analyses demonstrate that while often true, there is a need to look more deeply into HTS data to identify finer geographic areas where there could be higher numbers of HIV-infected persons than expected. Facility-level data are aggregated at an areal geographic unit for analyses. There is a lack of congruence in high targets based on the need to identify as many newly diagnosed HIV-infected persons in HIV high-burden areas as to the actual presence of potentially newly-infected persons. Thus, there is a need to rethink the target-setting approaches in clusters with the highest potential and refine geographical planning units to smaller units for better outcomes. Under targeting could have caused the lack of congruence between HTS targets and testing achievements where a higher number of HIV-infected persons were identified than was expected.

The role of transport infrastructure, including proximity and connectedness of road networks in HIV spread, has been described in sub-Saharan countries (15), and may also facilitate sex work (16). Good transportation networks may also be associated with the growing spread of HIV even in rural areas (14), and distance to urban centers may no longer be associated with prevalence hence the increasing rurality of HIV (17). We think that in areas with better road networks, access to services may have been a determinant for higher mobility, higher testing, and better identification of the number of newly diagnosed HIV-infected persons.

### Precision Targeting

Although access to HTS is needed everywhere in Kenya, intensive testing efforts can be complicated in low prevalence areas without identifying localized epidemics within larger geographical areas. A-priori facility-level targeting for the identification of HIV-infected persons is problematic for various reasons. Firstly, many individual client level and HTS access factors are difficult to consider in the targeting process. Secondly, previously undiscovered epidemic and new HIV diagnoses may occur in traditionally non-endemic regions. Such outbreaks may be driven by infrastructural developments such as road construction or high-volume economic activities. Distinct environments or geospatial features need to be considered for focusing HTS programs, including workplace HTS, HIV awareness, and prevention programs. Targeting requires that we take into account, not just the HIV burden but also spatial scale to increase precision. Geographically-oriented targeting and programming have been suggested by others (18, 19). However, putting such precision targeting into practice during COP requires timely analyses and good knowledge of the local context.

With constrained funding for home-based counseling and testing and other community-based testing approaches, it is imperative to focus HTS efforts geographically. Newer HTS approaches may improve the identification of newly diagnosed HIV-infected persons (20). Understanding individual-level characteristics that could be associated with HIV testing-seeking behavior is equally important. For example, using a household level survey, Waruru et al. found that economic status, perception of HIV risk, mobility, transactional sex, and uncircumcised men were associated with living in high HIV prevalence clusters in Kenya (8).

### Limitations

Our analyses had a few limitations. Firstly, we did not explore the spatial-temporal trend of HIV testing services or changing epidemiology of HIV, including transmission patterns over time due to the availability of data. Thus, our interpretation is limited to cross-sectional analyses and may not reflect the HIV program's historical investments in Kenya. Accounting for double reporting is difficult in Kenya because there are no unique healthcare identifiers to allow for an accurate account of duplicated results for both the number tested and the number diagnosed as HIV infected. Although our data were aggregated and did not explore individual characteristics of newly diagnosed HIV-infected persons, we could still get useful information for HIV program planning.

## Conclusions and Implications for HIV Epidemic Control

Using geospatial analyses and mapping, we have established and demonstrated that clusters with a high number of HIV-infected persons are in both high and low-burden counties. For Kenya to control the HIV epidemic, it is crucial to interrupt the spread of HIV from regions with an increased number of newly diagnosed HIV-infected persons that neighbor those with low numbers of HIV-infected persons. Having identified clusters where the number of newly diagnosed HIV-infected persons is higher, even within counties, these analyses demonstrate the need for micro-spatial analysis for efficient planning. Achieving the “first 90” will become a reality if HTS resources are redirected to areas with the highest potential for identifying HIV-infected persons who were not previously diagnosed. Cluster detection analysis can be useful in directing public health response by identifying areas that may have budding HIV infections, initiating rapid HTS, thereby contributing to accelerated testing and achievement of the “first 90.”

## Data Availability Statement

The raw data supporting this manuscript's conclusions will be made available by the authors, without undue reservation, to any qualified researcher.

## Author Contributions

AW conceived the idea for this manuscript, prepared the concept, data analyses, and wrote the first and subsequent drafts of the manuscript. JW, JM, EZ-G, LN, FM, PWY, KD, and TT helped with results interpretation provided insights on HIV programmatic implications and recommendations. TA and JT helped with analytic methods and data interpretation. PY helped with data cleaning. All authors read the manuscript, provided feedback, and approved the final version.

## Conflict of Interest

The authors declare that the research was conducted in the absence of any commercial or financial relationships that could be construed as a potential conflict of interest.
